# The metabotropic glutamate receptor 5 role on motor behavior involves specific neural substrates

**DOI:** 10.1186/s13041-015-0113-2

**Published:** 2015-04-10

**Authors:** Isabella M Guimaraes, Toniana G Carvalho, Stephen SG Ferguson, Grace S Pereira, Fabiola M Ribeiro

**Affiliations:** Departamento de Bioquimica e Imunologia, Instituto de Ciencias Biologicas, Universidade Federal de Minas Gerais, Belo Horizonte, 31270-901 Brazil; J. Allyn Taylor Centre for Cell Biology, Robarts Research Institute, University of Western Ontario, London, Ontario N6A 5 K8 Canada; Nucleo de Neurociencias, Departamento de Fisiologia e Biofisica, Instituto de Ciencias Biologicas, Universidade Federal de Minas Gerais, Belo Horizonte, 31270-901 Brazil

**Keywords:** mGluR5, Locomotor activity, Motor coordination, Striatum, Hippocampus

## Abstract

**Background:**

The metabotropic glutamate receptor 5 (mGluR5) is involved in various brain functions, including memory, cognition and motor behavior. Regarding locomotor activity, we and others have demonstrated that pharmacological antagonism of mGluR5 promotes hyperkinesia in mice. Moreover, increased locomotor activity can also be observed in mice following the genetic deletion of mGluR5. However, it is still unclear which specific brain substrates contribute to mGluR5-mediated regulation of motor function.

**Results:**

Thus, to better understand the role of mGluR5 in motor control and to determine which neural substrates are involved in this regulation we performed stereotactic microinfusions of the mGluR5 antagonist, MPEP, into specific brain regions and submitted mice to the open field and rotarod apparatus. Our findings indicate that mGluR5 blockage elicits distinct outcomes in terms of locomotor activity and motor coordination depending on the brain region injected with mGluR5 antagonist. MPEP injection into either the dorsal striatum or dorsal hippocampus resulted in increased locomotor activity, whereas MPEP injection into either the ventral striatum or motor cortex resulted in hypokinesia. Moreover, MPEP injected into the olfactory bulb increased the distance mice traveled in the center of the open field arena. With respect to motor coordination on the rotarod, injection of MPEP into the motor cortex and olfactory bulb elicited decreased latency to fall.

**Conclusions:**

Taken together, our data suggest that not only primarily motor neural substrates, but also limbic and sensory structures are involved in mGluR5-mediated motor behavior.

**Electronic supplementary material:**

The online version of this article (doi:10.1186/s13041-015-0113-2) contains supplementary material, which is available to authorized users.

## Background

Glutamate is the major excitatory neurotransmitter in the brain and is essential for a number of brain functions, including memory, cognition and neuronal cell development. Glutamate receptors are classified into two main families: ionotropic glutamate receptors, which are ligand-gated ion channels that mediate fast excitatory neurotransmission, and metabotropic glutamate receptors (mGluRs), which are members of the G protein-coupled receptor (GPCR) family [1-4]. There are three main types of ionotropic glutamate receptors, including N-methyl-D-aspartate receptor (NMDA), alpha-amino-3-hydroxy-5-methyl-4-isoxazolepropionic acid (AMPA), and kainate receptors [5,2]. The mGluR subfamily of GPCRs is comprised of eight different types of mGluRs that are sub-classified into three groups based on sequence homology and G protein specificity [1,6,3,4]. Group I mGluRs (mGluR1 and mGluR5) couple to Gα_q/11_ and promote the activation of phospholipase Cβ1, resulting in diacylglycerol and inositol-1,4,5-triphosphate formation, release of Ca^2+^ from intracellular stores and subsequent activation of protein kinase C. In contrast, group II (mGluR2 and mGluR3) and group III (mGluR4, mGluR6, mGluR7 and mGluR8) mGluRs inhibit adenylyl cyclase via Gα_i_.

mGluR5 protein and mRNA have been detected in various brain regions, including the olfactory bulb, cerebral cortex, hippocampus, lateral septum, striatum, nucleus accumbens, inferior colliculus, and spinal trigeminal nuclei [7,8]. Due to its widespread brain expression, mGluR5 is involved in various brain functions, including spontaneous locomotor activity and response to a new environment, as well as anxiety and cognitive functions such as spatial memory [9-12]. The role of mGluR5 in locomotor activity is well established, as we and others have demonstrated that pharmacological blockage of mGluR5 with either 2-methyl-6-[phenylethynyl]-pyridine (MPEP) or 3-[(2-methyl-4-thiazolyl)ethynyl]pyridine (MTEP) administered peripherally alter spontaneous locomotor activity and motor coordination in rodents [13,14]. Moreover, increased locomotor activity can also be observed in mGluR5 knockout mice [15,14]. However, it is still unclear which specific brain substrates are involved in mGluR5-mediated regulation of locomotor activity. It has been recently demonstrated that the knockout of mGluR5 exclusively in the cortex promotes increased novelty-induced locomotor activity [10]. Moreover, when these cortical-specific mGluR5 knockout mice were injected with MPEP intraperitoneally, they exhibited a pronounced increase in locomotor activity, which was much greater than that of wild type mice injected with MPEP [10]. These results indicate that the role of mGluR5 in locomotor activity might involve the cross-interaction of different neural substrates.

Thus, to better understand the role of mGluR5 in motor control and determine which neural substrates are involved in this regulation we performed stereotactic microinfusions of MPEP in select brain regions, including motor and parietal cortex, dorsal and ventral striatum, hippocampus and olfactory bulb, and submitted mice to the open field and rotarod apparatus. Our findings indicate that mGluR5 blockage elicit different outcomes in terms of locomotor activity and motor coordination depending on the brain area injected. Our data suggest that not only primarily motor neural substrates, but also limbic and sensory structures are involved in mGluR5-mediated motor behavior.

## Results

**Figure 1:**
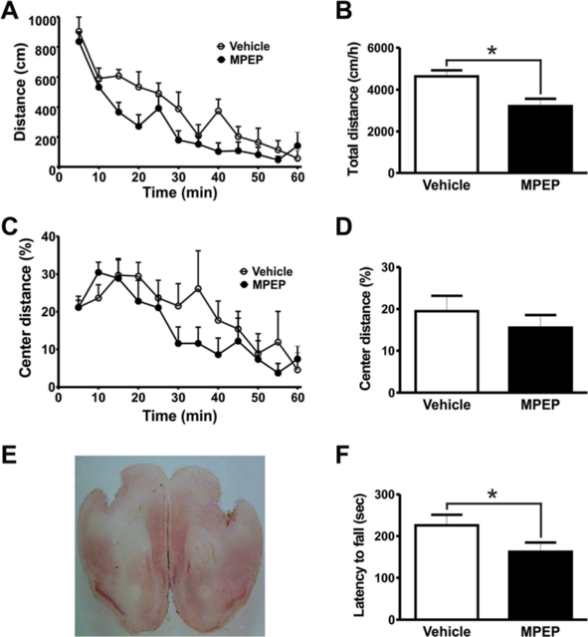
mGluR5 blockage in the primary motor cortex promotes reduction of both locomotor activity and rotarod performance. Graphs show distance traveled **(A)** and the percentage of distance traveled in the center of the apparatus **(C)** by mice injected with either vehicle (n = 7) or MPEP (n = 8) measured at 5 min intervals. Graphs show total distance traveled **(B)** and the percentage of distance traveled in the center **(D)** by mice injected with either vehicle (n = 7) or MPEP (n = 8) cumulative over 60 min. Animals were placed in the open field box after 10 min of either vehicle (DMSO 50%) or MPEP (25 nmol/0.5 μL/side) microinfusion into the primary motor cortex. Each animal was monitored for 60 min. **(E)** Shown is a photomicrography of a representative neutral red stained coronal brain section depicting guide cannula placement according to primary motor cortex coordinates. **(F)** Graph shows latency to fall from accelerating rotarod by mice injected with either vehicle (n = 7) or MPEP (25 nmol/0.5 μL/side) (n = 7). Each animal was tested in three trials and the average latency to fall was determined. Data represent the means ± SEM. ^*^ indicate significant differences as compared to vehicle-injected mice (P < 0.05).

In order to determine which brain substrates are involved in mGluR5-mediated motor control we performed stereotactic microinfusion of the mGluR5 negative allosteric modulator (NAM), MPEP, which is highly selective for mGluR5 [16], into specific brain coordinates targeting the various brain regions where the receptor is vastly expressed. First, we injected either MPEP 25 nmol/0.5 μL/side or vehicle into the primary motor cortex, which is a vital area for the regulation of locomotor activity, and 10 min later, subjected mice to the open field apparatus. Cannula placement into the primary motor cortex was confirmed by histology for all tested mice (Figure 1E). Statistical analyses (two-way ANOVA) indicated that mGluR5 blockage in the primary motor cortex caused a decrease in spontaneous locomotor activity when assessed in the open field apparatus [Interaction: *F*_(11,143)_ = 1.222, *P* = 0.2778; time: *F*_(11,143)_ = 24.05, *P* < 0.0001; treatment: *F*_(1,143)_ = 1.935, *P* = 0.0088] (Figure 1A). In addition, total distance traveled by mice injected with MPEP in the primary motor cortex was smaller than that of vehicle-injected mice [t_(13)_ = 3.077; *P* = 0.0044] (Figure 1B). It has been demonstrated that MPEP has anxiolytic-like effects when injected intraperitoneally [12]. Mice exhibiting increased anxiety tend to spend less time in the central area of the open field arena. Thus, we sought to determine whether injection of MPEP into the primary motor cortex would change the extent mice would walk in the central area of the arena. We found that the injection of mice with MPEP into the primary motor cortex did not increase the distance mice travelled in the center of the open field when compared with vehicle-injected mice [Interaction: *F*_(11,143)_ = 1.305, *P* = 0.2274; time: *F*_(11,143)_ = 9.124, *P <* 0.0001; treatment: *F*_(1,143)_ = 0.6899, *P* = 0.4212] (Figure 1C). Moreover, total distance traveled in the center was also not different between treatments [t_(13)_ = 0.8312; *P* = 0.2104] (Figure 1D), suggesting that this brain substrate may not be involved in mGluR5-mediated anxious behavior in the open field apparatus.

Following open field assessments, mice were trained on the rotarod for two days. On the third day, mice were microinfused with either MPEP or vehicle into the motor cortex and tested for motor coordination on the rotating rod. MPEP microinfusion in this brain substrate led to decreased latency to fall from the rotarod [t_(12)_ = 1.878; *P* = 0.0424] (Figure 1F). Together, these data indicate that the inhibition of mGluR5 expressed in the primary motor cortex inhibits locomotion and decreases motor coordination.

In order to further validate the inhibitory effect of mGluR5 blockage on locomotor activity and motor coordination, we performed microinfusions of another mGluR5 NAM, MTEP, into the primary motor cortex. Histological slices were analyzed to confirm cannula placement for all tested mice (Additional file 1: Figure S1E). Following injection of either MTEP 25 nmol/0.5 μL/side or vehicle into the primary motor area, mice were subjected to the open field apparatus. mGluR5 blockage by MTEP promoted decreased spontaneous locomotion activity when mice were submitted to the open field apparatus [Interaction: *F*_(11,110)_ = 1.848, *P* = 0.0543; time: *F*_(11,110)_ = 6.813, *P <* 0.0001; treatment: *F*_(1,110)_ = 14.51, *P* = 0.0034] (Additional file 1: Figure S1A) and [t_(10)_ = 3.809; *P* = 0.0017] (Additional file 1: Figure S1B). On the other hand, the distance mice traveled in the center of the arena was not significantly different when comparing mice injected with either vehicle or MTEP [Interaction: *F*_(11,99)_ = 1.660, *P* = 0.0938; time: *F*_(11,99)_ = 6.191, *P <* 0.0001; treatment: *F*_(1,99)_ = 9.512, *P* = 0.0131] (Additional file 1: Figure S1C) and [t_(10)_ = 1.197; *P* = 0.1295] (Additional file 1: Figure S1D). Furthermore, mice injected with MTEP into the primary motor cortex exhibited decreased latency to fall from the rotarod, as compared to that of vehicle-injected mice [t_(09)_ = 1.847; *P* = 0.0489] (Additional file 1: Figure S1F). These data demonstrate that the tested mGluR5 NAMs, MPEP and MTEP, promote decreased motor function when injected into the primary motor cortex and that either mGluR5 NAM can be used to assess mGluR5’s role on motor behavior.

**Figure 2:**
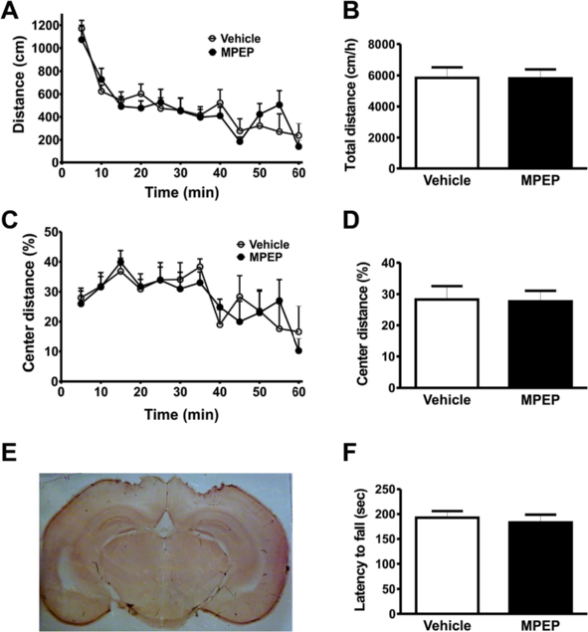
mGluR5 antagonism on posterior parietal cortex does not change locomotor activity or rotarod performance. Graphs show distance traveled **(A)** and the percentage of distance traveled in the center of the apparatus **(C)** by mice injected with either vehicle (n = 6) or MPEP (n = 6) measured at 5 min intervals. Graphs show total distance traveled **(B)** and the percentage of distance traveled in the center **(D)** by mice injected with either vehicle (n = 6) or MPEP (n = 6) cumulative over 60 min. Animals were placed in the open field box after 10 min of either vehicle (DMSO 50%) or MPEP (25 nmol/0.5 μL/side) microinfusion into the parietal cortex. Each animal was monitored for 60 min. **(E)** Shown is a photomicrography of a representative neutral red stained coronal brain section depicting guide cannula placement according to posterior parietal cortex coordinates. **(F)** Graph shows latency to fall from accelerating rotarod by mice injected with either vehicle (n = 6) or MPEP (25 nmol/0.5 μL/side) (n = 6). Each animal was tested in three trials and the average latency to fall was determined. Data represent the means ± SEM.

**Figure 3:**
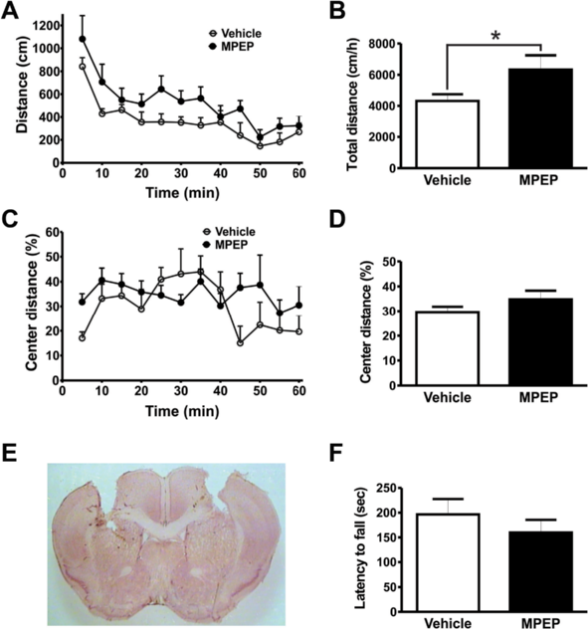
Acute antagonism of mGluR5 on dorsolateral striatum increases locomotor activity. Graphs show distance traveled **(A)** and the percentage of distance traveled in the center of the apparatus **(C)** by mice injected with either vehicle (n = 6) or MPEP (n = 6) measured at 5 min intervals. Graphs show total distance traveled **(B)** and the percentage of distance traveled in the center **(D)** by mice injected with either vehicle (n = 6) or MPEP (n = 6) cumulative over 60 min. Animals were placed in the open field box after 10 min of either vehicle (DMSO 50%) or MPEP (25 nmol/0.5 μL/side) microinfusion into the dorsolateral striatum. Each animal was monitored for 60 min. **(E)** Shown is a photomicrography of a representative neutral red stained coronal brain section depicting guide cannula placement according to dorsolateral striatum coordinates. **(F)** Graph shows latency to fall from accelerating rotarod by mice injected with either vehicle (n = 7) or MPEP (25 nmol/0.5 μL/side) (n = 7). Each animal was tested in three trials and the average latency to fall was determined. Data represent the means ± SEM. ^*^ indicates significant difference as compared to vehicle-injected mice (P < 0.05).

The posterior parietal cortex is an associative cortical brain region that controls visually guided movements and spatial orientation and expresses high levels of mGluR5 [17]. Thus, we decided to test whether MPEP microinfusion into the posterior parietal cortex could modify locomotor activity and motor coordination. We confirmed that cannula was placed in the right position (Figure 2E). Mice injected with MPEP in the posterior parietal cortex exhibited the same levels of locomotor activity as vehicle-treated mice [Interaction: *F*_(11,110)_ = 0.9575, *P* = 0.4893; time: *F*_(11,110)_ = 17.59, *P <* 0.0001; treatment: *F*_(1,110)_ = 0.01287, *P* = 0.9119] (Figure 2A) and [t_(10)_ = 0.03184; *P* = 0.4876] (Figure 2B). Moreover, distance traveled in the center of the arena was also not different between treatments [Interaction: *F*_(11,110)_ = 0.8268, *P* = 0.6136; time: *F*_(11,110)_ = 6.668, *P <* 0.0001; treatment: *F*_(1,110)_ = 0.01110, *P* = 0.9182] (Figure 2C) and [t_(10)_ = 0.1054; *P* = 0.4591] (Figure 3D). In addition, injection of MPEP into the posterior parietal cortex of mice did not increase the latency to fall from the rotarod as compared to vehicle-treated control mice [t_(10)_ = 0.4677; *P* = 0.3250] (Figure 2F). Thus, mGluR5 expressed in the posterior parietal cortex appears not to be involved in locomotor control.

**Figure 4:**
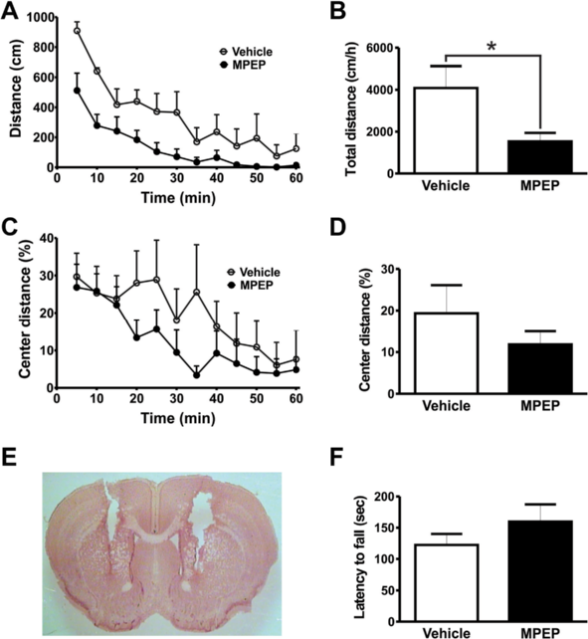
The inhibition of mGluR5 on ventral striatum promotes a reduction in locomotor activity. Graphs show distance traveled **(A)** and the percentage of distance traveled in the center of the apparatus **(C)** by mice injected with either vehicle (n = 6) or MPEP (n = 6) measured at 5 min intervals. Graphs show total distance traveled **(B)** and the percentage of distance traveled in the center **(D)** by mice injected with either vehicle (n = 6) or MPEP (n = 6) cumulative over 60 min. Animals were placed in the open field box after 10 min of either vehicle (DMSO 50%) or MPEP (25 nmol/0.5 μL/side) microinfusion into the ventral striatum. Each animal was monitored for 60 min. **(E)** Shown is a photomicrography of a representative neutral red stained coronal brain section depicting guide cannula placement according to ventral striatum coordinates. **(F)** Graph shows latency to fall from accelerating rotarod by mice injected with either vehicle (n = 8) or MPEP (25 nmol/0.5 μL/side) (n = 7). Each animal was tested in three trials and the average latency to fall was determined. Data represent the means ± SEM. ^*^ indicates significant difference as compared to vehicle-injected mice (P < 0.05).

mGluR5 is highly expressed in the striatum, which is an important region for motor control [18,19]. To investigate whether mGluR5 expressed in the striatum participates in movement regulation, we first performed stereotactic microinfusions of MPEP 25 nmol/0.5 μL/side into the dorsal striatum. Histological analyses indicated that cannula was correctly placed at the dorsal striatum (Figure 3E). Injection of MPEP into the dorsal striatum promoted a small increase in spontaneous locomotor activity, which did not reach statistical significance [Interaction: *F*_(11,110)_ = 0.6389, *P* = 0.7920; time: *F*_(11,110)_ = 13.20, *P* < 0.0001; treatment: *F*_(1,110)_ = 4.018, *P* = 0.0728] (Figure 3A). However, total distance traveled by animals injected with MPEP into the dorsal striatum was significantly greater than that of animals injected with vehicle [t_(10)_ = 2.005; *P* = 0.0364] (Figure 3B). We observed no MPEP-induced difference in the distance mice traveled in the center of the open field [Interaction: *F*_(11,110)_ = 1.533, *P* = 0.1297; time: *F*_(11,110)_ = 2.147, *P* = 0.0225; treatment: *F*_(1,110)_ = 1.572, *P* = 0.2385] (Figure 3C) and [t_(10)_ = 1.254; *P* = 0.1192] (Figure 3D) and MPEP treatment did not alter the latency to fall from the rotarod [t_(12)_ = 0.9155; *P* = 0.1890] (Figure 3F). In contrast, animals injected with MPEP into the ventral striatum (Figure 4E) demonstrated significantly reduced locomotor activity when compared to vehicle-treated control mice [Interaction: *F*_(11,110)_ = 1.675, *P* = 0.0885; time: *F*_(11,110)_ = 24.75, *P <* 0.0001; treatment: *F*_(1,110)_ = 5.261, *P* = 0.0447] (Figure 4A) and [t_(10)_ = 2.294; *P* = 0.0224] (Figure 4B). However, similar to what we observed for MPEP treatment of mice in the dorsal striatum, MPEP injection into the ventral striatum did not modify distance mice traveled in the center of the open field [Interaction: *F*_(11,110)_ = 1.135, *P* = 0.3414; time: *F*_(11,110)_ = 6.729, *P <* 0.0001; treatment: *F*_(1,110)_ = 0.9410, *P* = 0.3549] (Figure 4C) and [t_(10)_ = 1.005; *P* = 0.1693] (Figure 4D). Furthermore, microinfusion of MPEP into the ventral striatum did not alter performance on the rotarod apparatus [t_(13)_ = 1.168; *P* = 0.1318] (Figure 4F). Thus, depending on which region of the striatum mGluR5 is blocked, the result can be either hypokinesia or hyperkinesia.

**Figure 5:**
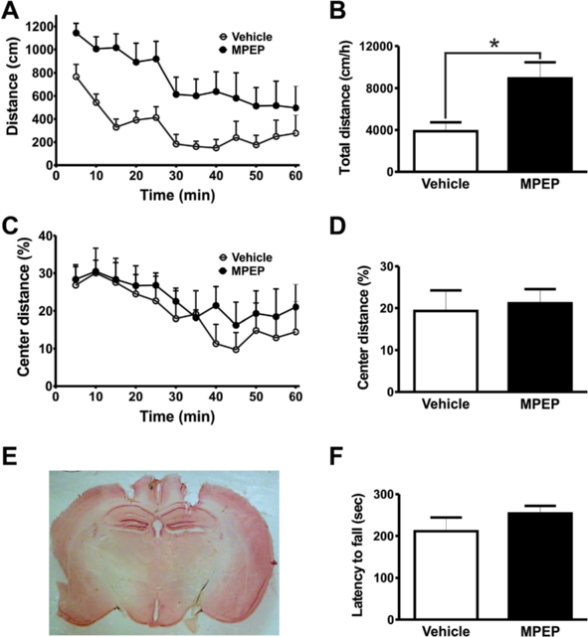
MPEP acute antagonism on dorsal hippocampus induces increased locomotor activity. Graphs show distance traveled **(A)** and the percentage of distance traveled in the center of the apparatus **(C)** by mice injected with either vehicle (n = 6) or MPEP (n = 7) measured at 5 min intervals. Graphs show total distance traveled **(B)** and the percentage of distance traveled in the center **(D)** by mice injected with either vehicle (n = 6) or MPEP (n = 7) cumulative over 60 min. Animals were placed in the open field box after 10 min of either vehicle (DMSO 50%) or MPEP (25 nmol/0.5 μL/side) microinfusion into the hippocampus. Each animal was monitored for 60 min. **(E)** Shown is a photomicrography of a representative neutral red stained coronal brain section depicting guide cannula placement according to dorsal hippocampus coordinates. **(F)** Graph shows latency to fall from accelerating rotarod by mice injected with either vehicle (n = 6) or MPEP (25 nmol/0.5 μL/side) (n = 7). Each animal was tested in three trials and the average latency to fall was determined. Data represent the means ± SEM. ^*^ indicates significant difference as compared to vehicle-injected mice (P < 0.05).

mGluR5 is also expressed at high levels in the hippocampus, a brain region that is involved in cognitive functions [20]. However, a number of publications indicate that this brain structure is also implicated in movement control [21,22]. Thus, we tested whether antagonism of mGluR5 activity in the dorsal hippocampus might alter locomotor activity. MPEP microinfusion into the dorsal hippocampus was confirmed by assessing cannula placement by histology (Figure 5E). MPEP injection into the dorsal hippocampus resulted in significantly increased motor activity of treated mice when compared to vehicle-treated controls [Interaction: *F*_(11,121)_ = 0.9099, *P* = 0.5332; time: *F*_(11,121)_ = 9.364, *P <* 0.0001; treatment: *F*_(1,121)_ = 7.643, *P* = 0.0184] (Figure 5A) and [t_(11)_ = 2.765; *P* = 0,0092] (Figure 5B). However, the distance traveled in the center of the open field arena was not affected by injection of MTEP into the dorsal hippocampus [Interaction: *F*_(11,121)_ = 0.3386, *P* = 0.9753, time: *F*_(11,121)_ = 4.472, *P <* 0.0001, treatment: *F*_(1,121)_ = 0.4223; *P* = 0.5291] (Figure 5C) and [t_(10)_ = 1.005; *P* = 0.1693] (Figure 5D). Furthermore, MPEP microinfusion into the dorsal hippocampus did not alter latency time to fall for treated mice versus vehicle-treated control [t_(11)_ = 1.186, *P* = 0.1304] (Figure 5F). According to these data, mGluR5 expressed in the dorsal hippocampus appears to be involved in spontaneous locomotor activity.

**Figure 6:**
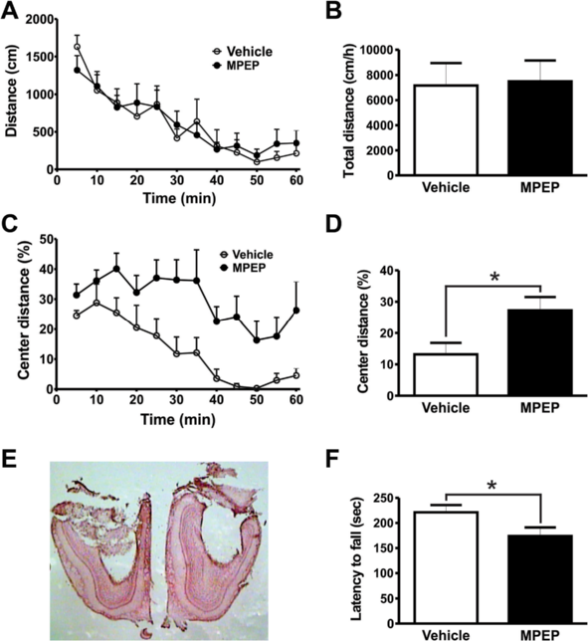
The focal inhibition of mGluR5 on the olfactory bulb alters locomotor performance and distance traveled in the center of the arena. Graphs show distance traveled **(A)** and the percentage of distance traveled in the center of the apparatus **(C)** by mice injected with either vehicle (n = 7) or MPEP (n = 8) measured at 5 min intervals. Graphs show total distance traveled **(B)** and the percentage of distance traveled in the center **(D)** by mice injected with either vehicle (n = 7) or MPEP (n = 8) cumulative over 60 min. Animals were placed in the open field box after 10 min of either vehicle (DMSO 50%) or MPEP (25 nmol/0.5 μL/side) microinfusion into the olfactory bulb. Each animal was monitored for 60 min. **(E)** Shown is a photomicrography of a representative neutral red stained coronal brain section depicting guide cannula placement according to olfactory bulb coordinates. **(F)** Graph shows latency to fall from accelerating rotarod by mice injected with either vehicle (n = 7) or MPEP (25 nmol/0.5 μL/side) (n = 7). Each animal was tested in three trials and the average latency to fall was determined. Data represent the means ± SEM. ^*^ indicate significant differences as compared to vehicle-injected mice (P < 0.05).

The olfactory bulb expresses high levels of mGluR5 and is a sensory brain area involved in olfaction [23]. Because the olfactory bulb is not known to be involved in motor control [24], as a control, we tested whether the antagonist of mGluR5 in the olfactory bulb would affect locomotor activity. Again, cannula placement was confirmed by histology (Figure 6E). As expected, spontaneous locomotor activity was not different when comparing mice that had the olfactory bulb microinfused with either MPEP or vehicle [Interaction: *F*_(11,143)_ = 0.8878, *P* = 0.5539, time: *F*_(11,143)_ = 24.34, *P <* 0.0001; treatment: *F*_(1,143)_ = 0.01356, *P* = 0.9091] (Figure 6A) and [t_(13)_ = 0.1383, *P* = 0.4461] (Figure 6B). However, surprisingly, injection of MPEP into the olfactory bulb significantly increased the distance mice traveled in the center, as compared to that of vehicle-injected mice [Interaction: *F*_(11,143)_ = 1.036, *P* = 0.4182, time: *F*_(11,143)_ = 8.539, *P <* 0.0001, treatment: *F*_(1,143)_ = 11.10; *P* = 0.0054] (Figure 6C) and [t_(13)_ = 2.459, *P* = 0.0144] (Figure 6D). Moreover, the latency to fall from the rotarod was reduced when mGluR5 was blocked in the olfactory bulb [t_(12)_ = 2.126, *P* = 0,0275] (Figure 6F). Together, these data suggest that mGluR5 expressed in the olfactory bulb could be important for motor coordination and anxiety behavior.

## Discussion

**Figure 7:**
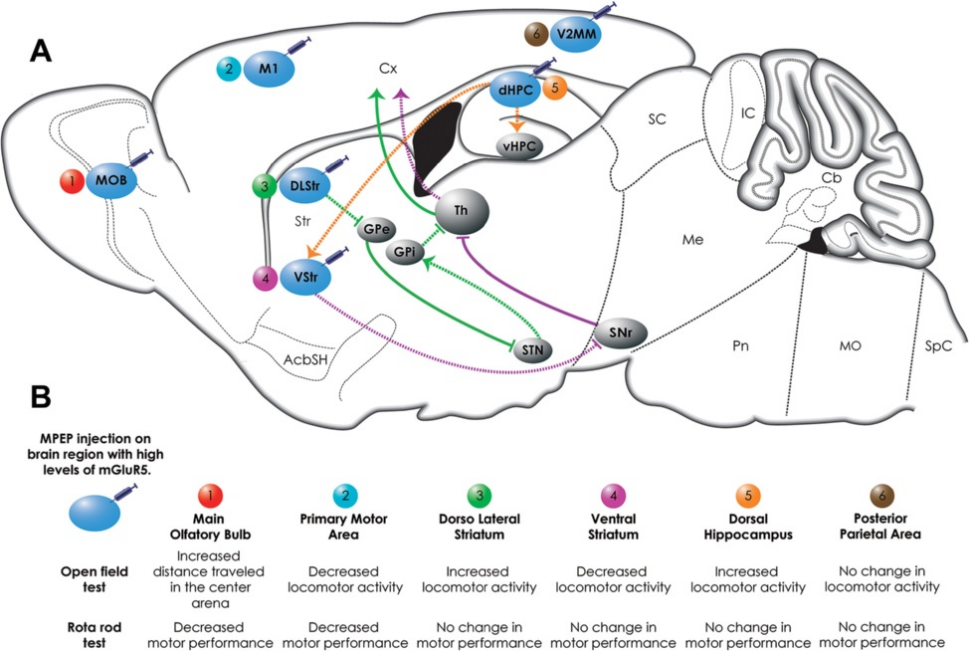
Potential neural pathways involved in motor behavior modulated by mGluR5. **(A)** Shows schematic representing CNS regions where MPEP injections were performed and the possible neural pathways involved in the behavioral findings following mGluR5 blockage. (1) MPEP injection on the main olfactory bulb (MOB) led to an increase in the distance traveled in the center of the arena and to decreased motor performance on the rotarod. (2) MPEP injection into the primary motor area (M1) led to a decrease in locomotion in the open field and to decreased rotarod performance. (3) The blockage of mGluR5 in the dorsolateral striatum (DLStr) led to increased locomotor activity. Inhibition of mGluR5 by MPEP in DLStr (*Green lines*) may disinhibit the globus pallidus externa (*GPe*), which can then inhibit the subthalamic nucleus (STN). STN inhibition will diminish activation of the substantia nigra pars reticulata (SNr)/ globus pallidus interna (GPi), with consequent disinhibition of the thalamus (Th) – cerebral cortical (Cx) circuit, resulting in increased locomotor activity. (4) The blockage of mGluR5 in the ventral striatum (VStr) by MPEP (*pink lines*) may disinhibit the SNr, which can inhibit Th-Cx projections, resulting in decreased locomotor activity. (5) mGluR5 inhibition in the dorsal hippocampus (dHPC) resulted in increased locomotor activity. The dHC projects to the VStr, which is involved in motor control (*orange lines*). Moreover, it has been shown that the dHPC has intrahippocampal projections connecting it to the ventral hippocampus (vHPC). (6) MPEP injections on the posterior parietal cortex (V2MM) elicited no alteration on behavioral tests. **(B)** Shows summary results of the behavioral findings for each neural substrate injected with MPEP. Blue circles indicate brain regions that highly express mGluR5 and that were injected with MPEP. Colorful lines (green, pink and orange) represent neural circuits. Filled lines indicate activated circuits and dotted lines indicate inhibited circuits. Excitatory pathways are depicted as arrows and inhibitory pathways as blocked lines.

It is well known that mGluR5 is involved in the regulation of locomotor activity, as it has been demonstrated that both mGluR5 pharmacological blockage and receptor knockout results in increased locomotor activity [15,13,14]. However, the specific brain regions regulating mGluR5-dependent alterations in locomotor activity have yet to de delineated. In the present study, we address that issue by performing stereotactic microinfusion of the mGluR5 antagonist, MPEP, into brain areas that express high levels of mGluR5, including the cortex, striatum, hippocampus and olfactory bulb (Figure 7), and assessing mouse motor behavior in the open field and rotarod apparatus. Our results indicate that the blockage of mGluR5 in neural substrates that are primary motor, as well as in limbic and sensory structures, can promote alterations in locomotor activity and motor coordination and balance.

mGluR5 is highly expressed in the brain and its activation promotes excitation, as this receptor signals through G_αq/11_, promoting activation of PKC and release of Ca^2+^ from intracellular stores, as well as positively modulating excitatory ion channels [5,7,8]. However, results from our group and others indicate that mGluR5 blockage induces hyperkinesia [13,14]. In addition to that, mGluR5 knockout mice exhibit a robust hyperkinetic phenotype [14,15]. Moreover, mGluR5 knockout exclusively in the cortex exhibit increased locomotor activity [10]. Interestingly, when we blocked mGluR5 with MPEP in different areas of the cortex, such as motor and parietal cortex, we obtained opposing results: decreased locomotor activity in the case of MPEP injection into the primary motor cortex and no change in locomotion in the case of injection into the posterior parietal cortex. Thus, mGluR5 blockage in different cortical sub-areas can elicit different motor outcomes, which could be due to the neural connections that these brain areas establish. Moreover, mGluR5 is expressed not only in glutamatergic neurons, but also in inhibitory neurons and glia [25,26]. Thus, it is possible that the antagonism of mGluR5 in the whole brain results in increased locomotor activity due to the interplay of the various neural circuitries. Interestingly, from our data it is clear that even regions that are not primary motor substrates, but limbic and sensory structures, appear to affect locomotor activity via mGluR5, which highlights the complexity of the neural networks that results in a specific animal behavior such as movement. Furthermore, there are other neural substrates that are important for motor control regulation, including the cerebellum, which has a crucial role related to balance and locomotion, and that do not express mGluR5 [8]. In this case, the underlying mechanism might not involve mGluR5 activation, but could involve other receptors also important for motor regulation [27].

The antagonist of mGluR5 activity in the dorsolateral striatum promoted increased locomotor activity, whereas receptor blockage in the ventral striatum decreased locomotor activity. These data further highlight how region-specific are the actions of mGluR5 to regulate movement. The region of the ventral striatum analyzed in this study corresponds to the nucleus accumbens core, which mainly receives input from the hippocampus, amygdala and prefrontal cortex, playing an important role in motivation and reward processes, as well as in locomotion [28,29]. The ventral striatum projects mainly to the dorsolateral part of the substantia nigra pars reticulata (SNr), to the ventral tegmental area (VTA) and to the substantia nigra pars compacta (SNc) [19]. Thus, inhibition of mGluR5 in the ventral striatum could: 1) disinhibit the SNc, which can stimulate (dopaminergic projection) the motor cortex and modulate the striatum; and 2) disinhibit the SNr, which can inhibit (GABAergic projection) thalamus-cortex [30]. Our results showing that MPEP injection into the ventral striatum diminishes locomotor activity are in agreement with the hypothesis that mGluR5 may be involved in the ventral striatum output to the SNr (Figure 7). The dorsal striatum, on the other hand, corresponds to the caudate and putamen in primates and closely regulates sensorimotor behavior [31]. The striatum dorsal portion can be further divided into two sub-regions: 1) an external portion (dorsolateral), which corresponds to the primate putamen and receives projections from the sensorimotor cortex and thalamus, and 2) an internal portion (dorsomedial), which is homologous to the primate caudate and receives projections from the prefrontal and associative cortex, amygdala and hippocampus [32,19]. Functionally, the dorsalmedial striatum is more similar to the ventral striatum. The dorsolateral area, which is the main striatal area involved in motor function, projects to the ventrolateral SNr [33]. Thus, mGluR5 inhibition in the dorsolateral striatum could disinhibit the SNr, which can promote thalamo-cortical inhibition. However, another dorsolateral striatum output target is the globus pallidus externa (GPe). In this case, MPEP injection into the dorsolateral striatum could disinhibit the GPe, which then can inhibit the subthalamic nucleus (STN) that will diminish activation of the SNr/globus pallidus interna (GPi) with consequent decrease in thalamus-cortical inhibition (Figure 7) [30]. In this case the result would be increased locomotor activity, as found in our current study. However, there are many intrinsic circuits within the basal ganglia and a much higher level of complexity in terms of the variety of neurotransmitters involved in locomotor activity. Therefore, additional experimentation focusing on the role of other neurotransmitters, such as acetylcholine and dopamine, will be necessary to determine which circuits and brain areas are underlying the locomotor alterations observed when MPEP was injected in the ventral and dorsal striatum.

Our data indicate that mGluR5 blockage in the hippocampus, which is a brain substrate well known for its role on memory and cognition [34,20], produces hyperkinesia. One potential hypothesis to explain this observation is that hyperkinesia following hippocampal mGluR5 blockage might reflect a deficit in the animal’s habituation to the environment due to disruption of hippocampal-dependent spatial and contextual memory [35,36]. However, this hypothesis does not adequately explain our current findings, as a habituation deficit would be reflected by a delayed onset of hyperactivity and our data demonstrated that hippocampal injection of MPEP led to immediate hyperactivity (Figure 5A). Another possibility is that mGluR5 blockage in the hippocampus modulates locomotor activity directly. The hippocampus can be divided into dorsal and ventral hippocampus [20,19]. In terms of function, the dorsal hippocampus appears to be involved in spatial learning and memory, whereas the ventral hippocampus is important for motor functions, as it directly connects to the prefrontal cortex, amygdala and ventral striatum (nucleus accumbens) [37,38]. However, the dorsal hippocampus also exhibit an output to the ventral striatum (Figure 7) [39], which could implicate this neural substrate in motor control. Moreover, there are intrahippocampal projections connecting dorsal and ventral hippocampus [40], which implies that the ventral role on motor modulation can be influenced by dorsal hippocampus manipulation (Figure 7). Supporting this hypothesis, a previous study demonstrated that an ischemic insult that promotes loss of 80% of the dorsal hippocampus (CA1 region) elicits hyperkinesia in rodents [21]. Moreover, microinfusion of the NMDA receptor antagonist, MK801, into the ventral and dorsal hippocampus promotes increased locomotor activity in the open field apparatus, although the increase promoted by dorsal hippocampus MK801 infusion was lesser than that of ventral infusion [41,42]. The hyperactivity following hippocampal lesions has been proposed to reflect the loss of inhibitory control over the dopaminergic tonus in the ventral striatum [43]. In agreement with this idea, our results demonstrate that MPEP injection into the dorsal hippocampus promoted increased locomotor activity, whereas injection of MPEP into the ventral striatum elicited hypokinesia.

To our surprise, injection of MPEP into the olfactory bulb, which is a primary olfactory sensory region of the brain, led to decreased latency to fall from the rotarod and increased distance traveled in the center of the open field arena. Nevertheless, it has been demonstrated that olfactory bulbectomy can alter exploratory behavior, locomotor activity and social interaction [44,45]. Moreover, different authors have reported that olfactory bulbectomy increases anxiety and that anxiolytic drugs normalize this behavior [44,46,47]. However, when submitted to the social interaction test, which is a well-accepted paradigm to measure anxiety, olfactory bulbectomized mice exhibited increased social interaction [44]. It is well established that intraperitoneally-injected MPEP has anxiolytic-like effects [12] and our data indicate that MPEP injection into the olfactory bulb seems to decrease anxiety, as mice spent more time in the center of the arena. These data indicate that mGluR5 might have a role in anxious behavior via olfactory bulb. However, the distance traveled in the center of the arena per se is not enough to determine whether MPEP is capable of decreasing anxiety via olfactory bulb and additional anxiety tests will be required to further investigate this issue.

## Conclusions

In conclusion, our results highlight the importance of mGluR5 in modulating motor behavior in a variety of brain regions. Our data indicate that regulation of locomotor activity seems to involve well known primary motor brain areas, as well as somatosensory and limbic brain structures (Figure 7). Moreover, to our surprise, brain substrates that are important for mGluR5-mediated regulation of locomotor activity appears to differ from those that modulate motor coordination via mGluR5. For instance, the only MPEP-injected brain region that exhibited both locomotor activity and rotarod alterations was the primary motor cortex. Interestingly, although it is very clear from the literature that the striatum is important for motor coordination [48,49], MPEP injection into the dorsal and ventral striatum did not modify rotarod performance, even though it altered locomotor activity. Thus, our data support the idea that dissecting the neural circuits involved in mGluR5-mediated motor behavior regulation is very important to better understand the physiological role of this receptor, as well as its role in a number of diseases that involve motor alterations, including Huntington’s disease. Future studies will be necessary to further elucidate the mGluR5-realated connections among the different brain substrates.

## Methods

### Materials

2-methyl-6-(phenylethynyl)-pyridine (MPEP) and 3-[(2-methyl-1,3-thiazol-4-yl) ethynyl] pyridine (MTEP) were purchased from Tocris Bioscience (Bristol, UK). Dimethyl sulfoxide (DMSO) and paraformaldehyde were purchased from Sigma Aldrich (St. Louis, MO, U.S.A.). Neutral red A.R. was purchased from Himedia Laboratories (Mumbai, MH, India). Glass microscope slides (25,4 mm x 76,2 mm) were from Global Glass (Beilun, ZHE, China). Sucrose was purchased from Synth (Diadema, SP, Brazil) and saline solution 0.9% (NaCl) from Equiplex (Aparecida de Goiânia, GO, Brazil). Ketamine hydrochloride and xylazine hydrochloride were purchased from Syntec (Cotia, SP, Brazil) and flunixin meglumine - Banamine® from Schering-Plough (Kenilworth, NJ, U.S.A). The zinc cement and dental acrylic were purchased from Coltene (São Jose, SC, Brazil) and cephalexin from Medley (Brasilia, DF, Brazil). The polyethylene tubing (PE20) was purchased from Tygon® Tubing (Ohio, U.S.A.).

### Animals

This study was conducted using male C57/BL6 mice (25-30 g) that were purchased from the animal facility (CEBIO) located at the Universidade Federal de Minas Gerais (UFMG). Mice were housed in an animal care facility at 23°C on a 12 h light/12 h dark cycle with food and water provided *ad libitum*. The number of mice, with correctly placed cannula, used for the primary motor cortex injection experiments was 27 (7 vehicle and 8 MPEP and 6 vehicle and 6 MTEP), for the posterior parietal cortex it was 12 mice (6 vehicle and 6 MPEP), for the dorsolateral striatum it was 14 mice (7 vehicle and 7 MPEP), for the ventral striatum it was 15 mice (8 vehicle and 7 MPEP), for the dorsal hippocampus it was 13 mice (6 vehicle and 7 MPEP) and for olfactory bulb it was 15 mice (7 vehicle and 8 MPEP). The experimental procedures used in this study received approval from CETEA-UFMG (Ethics Committee for Animal Experimentation - UFMG), protocol #274/2011.

### Drugs

MPEP was diluted in vehicle consisting of 50% dimethyl sulfoxide (DMSO) in saline (0.9% NaCl) and MTEP was diluted in vehicle consisting of saline only. Each neural structure was microinfused with either vehicle, 25 nmol MPEP or 25 nmol MTEP, in a final volume of 0.5 μL per/side, which is in accordance with published data [50-53].

### Cannula implantation and microinfusion

**Figure 8:**
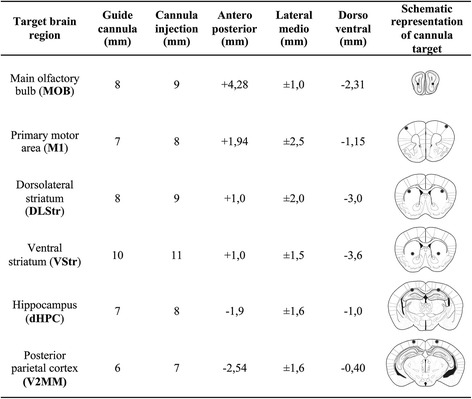
Description of stereotaxic coordinates from bregma and features of the guide cannula and injector.

Surgery procedures were performed according to [54]. Mice were not handled before surgery. Before surgery procedures, animals were anesthetized with ketamine (80 mg/kg) and xylazine (10 mg/kg) intraperitoneally (i.p.) and placed in the stereotaxic apparatus (Stoelting, Italy). Bregma and lambda were aligned at the same horizontal and vertical planes. Small holes (0.7 mm) were drilled directed into the skull according to the stereotaxic coordinates (Figure 8). Bilateral guide cannulae with metal occluding rods were fixed into the skull with zinc cement followed by dental acrylic. Following cannula implantation, mice received a single intramuscular dose of flunixin meglumine (Banamine®, 0.3 mg/kg) and two doses of cephalexin (72 mg/kg) orally. Time of post-surgical recovery was 4 to 5 days before the start of behavioral testing. To perform drug microinfusions, mice were gently immobilized and positioned for removal of the metal occluding rods and coupling of an injector cannula (30G, 8 mm) to the guide cannula. All drugs were infused in a volume of 0.5 μL/side. The microinfusion pump was made through a polyethylene tubing (PE20) connected to a 10 μL syringe (Hamilton, U.S.A.). Drugs were injected bilaterally in specific brain regions (Figure 8) at a rate of 0.5 μL/min. Injector cannula remained in place for 1 min after infusion to avoid diffusion of the drug through the guide cannula. Animals were subjected to behavioral tests 10 min after removal of the injection cannula.

### Histology

At the end of the behavioral experiments, mice were euthanized and their brains were removed and stored in 10% paraformaldehyde for two days, followed by three days in 30% sucrose. 100 μm thick coronal sections were obtained using cryostat (−20°C). Slices were stained with neutral red and visualized by light microscopy. Only mice with correct placement of cannula were included in statistical analyses.

### Open field test

Spontaneous locomotor activity was assessed using an automatic open field apparatus (LE 8811 IR Motor Activity Monitors PANLAB, Harvard Apparatus; Spain), with acrylic box dimension of 450 x 450 x 200 mm (width x depth x height). All experiments using open field apparatus were performed during the light cycle. First, mice were habituated to the behavioral testing room for at least 60 min. 10 min after drug infusion, animals were placed in the open field apparatus and the horizontal activity (distance traveled) was measured during 60 min. Quantification of the total activity was calculated using the ACTITRACK program.

### Rotarod test

Mice training and behavioral testing on the rotarod system (Insight, Ribeirao Preto, Brazil) were performed during the light cycle. Mice were habituated to the behavioral testing room for at least 60 min. Initially, animals were submitted to the training protocol on the rotarod for 2 days, when mice were placed on the rotating cylinder for 2 min in the first five speeds ranging from 5 to 19 rotations per min. On the third day, which was the testing day, mice were injected with either vehicle or drug and, 10 min later, placed on the rotarod apparatus. The acceleration protocol was performed in three independent experiments with an interval of 10 min between trials. If mice fell in the first 10 seconds, they were immediately relocated to the apparatus and count was restarted. The latency to fall from the rotating cylinder was recorded, and the average obtained from the three trials was used for analysis. The time limit for mice to remain on the rotarod was up to 300 seconds. After this period, mice were removed from the apparatus.

### Statistical analysis

Means ± SEM are shown for the number of animals indicated in figure legends. GraphPadPrism® version 5 (GraphPad Software Inc., San Diego, USA) software was used to analyze data for statistical significance and for curve fitting. Statistical significance was determined by analysis of variance (ANOVA, repeated measures) and Student’s t-test and were considered significant when p < 0.05.

## Additional file

Additional file 1: Figure S1. MTEP blockage of mGluR5 in the primary motor cortex reduces both locomotor activity and rotarod performance. Graphs show distance traveled **(A)** and the percentage of distance traveled in the center of the apparatus **(C)** by mice injected with either vehicle (n = 6) or MTEP (n = 6) measured at 5 min intervals. Graphs show total distance traveled **(B)** and the percentage of distance traveled in the center **(D)** by mice injected with either vehicle (n = 6) or MTEP (n = 6) cumulative over 60 min. Animals were placed in the open field box after 10 min of either vehicle (saline 0.9% NaCl) or MTEP (25 nmol/0.5 μL/side) microinfusion into the primary motor cortex. Each animal was monitored for 60 min. **(E)** Shown is a photomicrography of a representative neutral red stained coronal brain section depicting guide cannula placement according to primary motor cortex coordinates. **(F)** Graph shows latency to fall from accelerating rotarod by mice injected with either vehicle (n = 6) or MTEP (25 nmol/0.5 μL/side) (n = 5). Each animal was tested in three trials and the average latency to fall was determined. Data represent the means ± SEM. ^*****^ indicate significant differences as compared to vehicle-injected mice (P < 0.05).

## Abbreviations

AMPA: Alpha-amino-3-hydroxy-5methyl-4isoxazolepropionic acid
DMSO: Dimethyl sulfoxide
DLStr: Dorsolateral striatum
GABA: Gamma-aminobutyric acid
GPCR: G protein-coupled receptor
GPe: Globus pallidus externa
dHPC: dorsal hippocampus
GPi: Globus pallidus interna
i.p: Intraperitoneally
mGluR: metabotropic glutamate receptor
MOB: Main olfactory bulb
MPEP: 2-methyl-6-[phenylethynyl]-pyridine
MTEP: 3-[(2-methyl-4-thiazolyl)ethynyl]pyridine
NaCl: Sodium chloride
NAM: Negative allosteric modulator
NMDA: N-methyl-D-aspartate receptor
V2MM: Parietal associative area
M1: Primary motor area
PKC: Protein kinase C
SNc: Substantia nigra pars compacta
SNr: Substantia nigra pars reticulata
STN: Subthalamic nucleus
VStr: Ventral striatum
VTA: Ventral tegmental area

## References

[1] Dhami GK, Ferguson SS (2006). Regulation of metabotropic glutamate receptor signaling, desensitization and endocytosis. Pharmacol Ther.

[2] Dingledine R, Borges K, Bowie D, Traynelis SF (1999). The glutamate receptor ion channels. Pharmacol Rev.

[3] Pin JP, Galvez T, Prezeau L (2003). Evolution, structure, and activation mechanism of family 3/C G-protein-coupled receptors. Pharmacol Ther.

[4] Ribeiro FM, Paquet M, Cregan SP, Ferguson SS (2010). Group I metabotropic glutamate receptor signalling and its implication in neurological disease. CNS Neurol Disord Drug Targets.

[5] Conn PJ, Pin JP (1997). Pharmacology and functions of metabotropic glutamate receptors. Annu Rev Pharmacol Toxicol.

[6] Nakanishi S (1992). Molecular diversity of glutamate receptors and implications for brain function. Science.

[7] Kerner JA, Standaert DG, Penney JB, Young AB, Landwehrmeyer GB (1997). Expression of group one metabotropic glutamate receptor subunit mRNAs in neurochemically identified neurons in the rat neostriatum, neocortex, and hippocampus. Brain Res Mol Brain Res.

[8] Shigemoto R, Nomura S, Ohishi H, Sugihara H, Nakanishi S, Mizuno N (1993). Immunohistochemical localization of a metabotropic glutamate receptor, mGluR5, in the rat brain. Neurosci Lett.

[9] Balschun D, Zuschratter W, Wetzel W (2006). Allosteric enhancement of metabotropic glutamate receptor 5 function promotes spatial memory. Neuroscience.

[10] Jew CP, Wu CS, Sun H, Zhu J, Huang JY, Yu D (2013). mGluR5 ablation in cortical glutamatergic neurons increases novelty-induced locomotion. PLoS One.

[11] Kinney GG, Burno M, Campbell UC, Hernandez LM, Rodriguez D, Bristow LJ (2003). Metabotropic glutamate subtype 5 receptors modulate locomotor activity and sensorimotor gating in rodents. J Pharmacol Exp Ther.

[12] Tatarczynska E, Klodzinska A, Chojnacka-Wojcik E, Palucha A, Gasparini F, Kuhn R (2001). Potential anxiolytic- and antidepressant-like effects of MPEP, a potent, selective and systemically active mGlu5 receptor antagonist. Br J Pharmacol.

[13] McGeehan AJ, Janak PH, Olive MF (2004). Effect of the mGluR5 antagonist 6-methyl-2-(phenylethynyl)pyridine (MPEP) on the acute locomotor stimulant properties of cocaine, D-amphetamine, and the dopamine reuptake inhibitor GBR12909 in mice. Psychopharmacology.

[14] Ribeiro FM, Devries RA, Hamilton A, Guimaraes IM, Cregan SP, Pires RG (2014). Metabotropic glutamate receptor 5 knockout promotes motor and biochemical alterations in a mouse model of Huntington's disease. Hum Mol Genet.

[15] Gray L, van den Buuse M, Scarr E, Dean B, Hannan AJ (2009). Clozapine reverses schizophrenia-related behaviours in the metabotropic glutamate receptor 5 knockout mouse: association with N-methyl-D-aspartic acid receptor up-regulation. Int J Neuropsychopharmacol.

[16] Gasparini F, Lingenhohl K, Stoehr N, Flor PJ, Heinrich M, Vranesic I (1999). 2-Methyl-6-(phenylethynyl)-pyridine (MPEP), a potent, selective and systemically active mGlu5 receptor antagonist. Neuropharmacology.

[17] Calton JL, Taube JS (2009). Where am I and how will I get there from here? A role for posterior parietal cortex in the integration of spatial information and route planning. Neurobiol Learn Mem.

[18] Reiner A, Medina L, Veenman CL (1998). Structural and functional evolution of the basal ganglia in vertebrates. Brain Res Brain Res Rev.

[19] Voorn P, Vanderschuren LJ, Groenewegen HJ, Robbins TW, Pennartz CM (2004). Putting a spin on the dorsal-ventral divide of the striatum. Trends Neurosci.

[20] Fanselow MS, Dong HW (2010). Are the dorsal and ventral hippocampus functionally distinct structures?. Neuron.

[21] Kuroiwa T, Bonnekoh P, Hossmann KA (1991). Locomotor hyperactivity and hippocampal CA1 injury after transient forebrain ischemia of gerbils. Neurosci Lett.

[22] Zhang WN, Bast T, Feldon J (2002). Effects of hippocampal N-methyl-D-aspartate infusion on locomotor activity and prepulse inhibition: differences between the dorsal and ventral hippocampus. Behav Neurosci.

[23] Auffarth B (2013). Understanding smell–the olfactory stimulus problem. Neurosci Biobehav Rev.

[24] Imai T (2014). Construction of functional neuronal circuitry in the olfactory bulb. Semin Cell Dev Biol.

[25] Aronica E, Gorter JA, Ijlst-Keizers H, Rozemuller AJ, Yankaya B, Leenstra S (2003). Expression and functional role of mGluR3 and mGluR5 in human astrocytes and glioma cells: opposite regulation of glutamate transporter proteins. Eur J Neurosci.

[26] Testa CM, Standaert DG, Landwehrmeyer GB, Penney JB, Young AB (1995). Differential expression of mGluR5 metabotropic glutamate receptor mRNA by rat striatal neurons. J Comp Neurol.

[27] Morton SM, Bastian AJ (2004). Cerebellar control of balance and locomotion. Neurosci Rev J Bring Neurobiol Neurol Psychiatry.

[28] Kakei S, Hoffman DS, Strick PL (1999). Muscle and movement representations in the primary motor cortex. Science.

[29] Robbins TW, Everitt BJ (1996). Neurobehavioural mechanisms of reward and motivation. Curr Opin Neurobiol.

[30] Smith Y, Bevan MD, Shink E, Bolam JP (1998). Microcircuitry of the direct and indirect pathways of the basal ganglia. Neuroscience.

[31] Groenewegen HJ (2003). The basal ganglia and motor control. Neural Plasticity.

[32] Graybiel AM (1991). Basal ganglia–input, neural activity, and relation to the cortex. Curr Opin Neurobiol.

[33] Maurin Y, Banrezes B, Menetrey A, Mailly P, Deniau JM (1999). Three-dimensional distribution of nigrostriatal neurons in the rat: relation to the topography of striatonigral projections. Neuroscience.

[34] Bast T, Feldon J (2003). Hippocampal modulation of sensorimotor processes. Prog Neurobiol.

[35] Anagnostaras SG, Maren S, Fanselow MS (1999). Temporally graded retrograde amnesia of contextual fear after hippocampal damage in rats: within-subjects examination. J Neurosci Off J Soc Neurosci.

[36] Fanselow MS (2000). Contextual fear, gestalt memories, and the hippocampus. Behav Brain Res.

[37] Moser E, Moser MB, Andersen P (1993). Spatial learning impairment parallels the magnitude of dorsal hippocampal lesions, but is hardly present following ventral lesions. J Neurosci Off J Soc Neurosci.

[38] Wu M, Brudzynski SM (1995). Mesolimbic dopamine terminals and locomotor activity induced from the subiculum. Neuroreport.

[39] Groenewegen HJ, Vermeulen Van Der Zee E, te Kortschot A, Witter MP (1987). Organization of the projections from the subiculum to the ventral striatum in the rat. A study using anterograde transport of Phaseolus vulgaris leucoagglutinin. Neuroscience.

[40] Amaral DG, Witter MP (1989). The three-dimensional organization of the hippocampal formation: a review of anatomical data. Neuroscience.

[41] Zhang WN, Bast T, Feldon J (2000). Microinfusion of the non-competitive N-methyl-D-aspartate receptor antagonist MK-801 (dizocilpine) into the dorsal hippocampus of wistar rats does not affect latent inhibition and prepulse inhibition, but increases startle reaction and locomotor activity. Neuroscience.

[42] Zhang WN, Bast T, Feldon J (2001). The ventral hippocampus and fear conditioning in rats: different anterograde amnesias of fear after infusion of N-methyl-D-aspartate or its noncompetitive antagonist MK-801 into the ventral hippocampus. Behav Brain Res.

[43] Coutureau E, Galani R, Jarrard LE, Cassel JC (2000). Selective lesions of the entorhinal cortex, the hippocampus, or the fimbria-fornix in rats: a comparison of effects on spontaneous and amphetamine-induced locomotion. Exp Brain Res.

[44] Flores G, Ibanez-Sandoval O, Silva-Gomez AB, Camacho-Abrego I, Rodriguez-Moreno A, Morales-Medina JC (2014). Neonatal olfactory bulbectomy enhances locomotor activity, exploratory behavior and binding of NMDA receptors in pre-pubertal rats. Neuroscience.

[45] Kelly JP, Wrynn AS, Leonard BE (1997). The olfactory bulbectomized rat as a model of depression: an update. Pharmacol Ther.

[46] Kamei J, Hirose N, Oka T, Miyata S, Saitoh A, Yamada M (2007). Effects of methylphenidate on the hyperemotional behavior in olfactory bulbectomized mice by using the hole-board test. J Pharmacol Sci.

[47] Wang D, Noda Y, Tsunekawa H, Zhou Y, Miyazaki M, Senzaki K (2007). Behavioural and neurochemical features of olfactory bulbectomized rats resembling depression with comorbid anxiety. Behav Brain Res.

[48] Knowlton BJ, Mangels JA, Squire LR (1996). A neostriatal habit learning system in humans. Science.

[49] Ogura T, Ogata M, Akita H, Jitsuki S, Akiba L, Noda K (2005). Impaired acquisition of skilled behavior in rotarod task by moderate depletion of striatal dopamine in a pre-symptomatic stage model of Parkinson's disease. Neurosci Res.

[50] Simonyi A, Serfozo P, Parker KE, Ramsey AK, Schachtman TR (2009). Metabotropic glutamate receptor 5 in conditioned taste aversion learning. Neurobiol Learn Mem.

[51] Molina-Hernandez M, Tellez-Alcantara NP, Perez-Garcia J, Olivera-Lopez JI, Jaramillo MT (2006). Antidepressant-like and anxiolytic-like actions of the mGlu5 receptor antagonist MTEP, microinjected into lateral septal nuclei of male Wistar rats. Prog Neuro-Psychopharmacol Biol Psychiatry.

[52] Martinez-Rivera A, Rodriguez-Borrero E, Matias-Aleman M, Montalvo-Acevedo A, Guerrero-Figuereo K, Febo-Rodriguez LJ (2013). Metabotropic glutamate receptor 5 within nucleus accumbens shell modulates environment-elicited cocaine conditioning expression. Pharmacol Biochem Behav.

[53] Phillips JM, Lam HA, Ackerson LC, Maidment NT (2006). Blockade of mGluR glutamate receptors in the subthalamic nucleus ameliorates motor asymmetry in an animal model of Parkinson's disease. Eur J Neurosc.

[54] Pereira LM, Bastos CP, de Souza JM, Ribeiro FM, Pereira GS (2014). Estradiol enhances object recognition memory in Swiss female mice by activating hippocampal estrogen receptor alpha. Neurobiol Learn Mem.

