# Supplementary figures and images for: The metabotropic glutamate receptor 5 role on motor behavior involves specific neural substrates
Source: Mol Brain. 2015 Apr 10;8:24. doi: 10.1186/s13041-015-0113-2 (PMC4397819; doi:10.1186/s13041-015-0113-2)

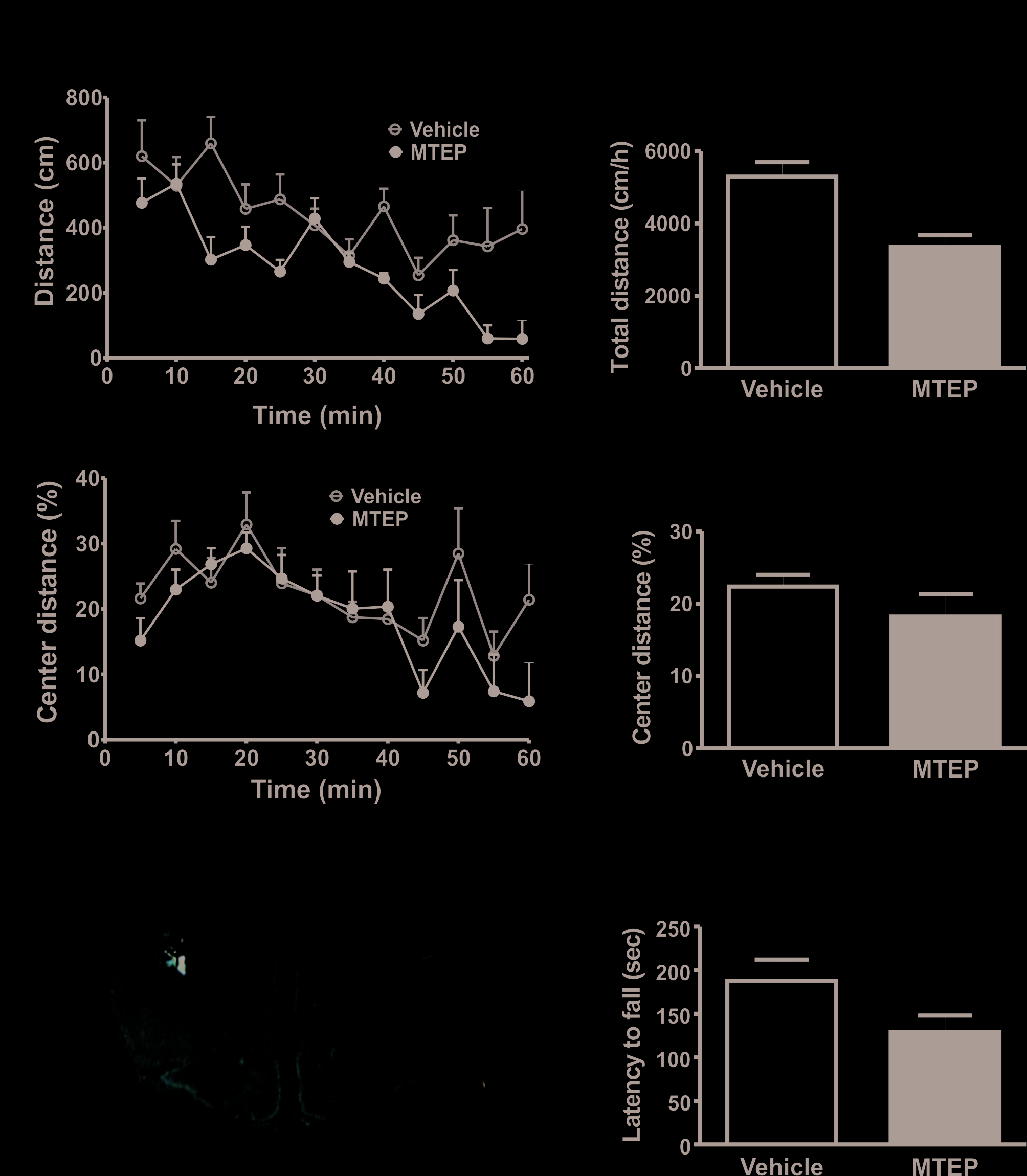

Supplement: Additional file 1: Figure S1. — MTEP blockage of mGluR5 in the primary motor cortex reduces both locomotor activity and rotarod performance. Graphs show distance traveled (A) and the percentage of distance traveled in the center of the apparatus (C) by mice injected with either vehicle (n = 6) or MTEP (n = 6) measured at 5 min intervals. Graphs show total distance traveled (B) and the percentage of distance traveled in the center (D) by mice injected with either vehicle (n = 6) or MTEP (n = 6) cumulative over 60 min. Animals were placed in the open field box after 10 min of either vehicle (saline 0.9% NaCl) or MTEP (25 nmol/0.5 μL/side) microinfusion into the primary motor cortex. Each animal was monitored for 60 min. (E) Shown is a photomicrography of a representative neutral red stained coronal brain section depicting guide cannula placement according to primary motor cortex coordinates. (F) Graph shows latency to fall from accelerating rotarod by mice injected with either vehicle (n = 6) or MTEP (25 nmol/0.5 μL/side) (n = 5). Each animal was tested in three trials and the average latency to fall was determined. Data represent the means ± SEM. * indicate significant differences as compared to vehicle-injected mice (P < 0.05). [file 13041_2015_113_MOESM1_ESM.tiff]
